# Social Networking in Adolescents: Time, Type and Motives of Using, Social Desirability, and Communication Choices

**DOI:** 10.3390/ijerph19042418

**Published:** 2022-02-19

**Authors:** Marta Tremolada, Lucio Silingardi, Livia Taverna

**Affiliations:** 1 Department of Developmental and Social Psychology, University of Padua, 35131 Padua, Italy; lucio.silingardi@gmail.com; 2 Faculty of Education, Free University of Bolzano-Bozen, 39042 Brixen, Italy; livia.taverna@unibz.it

**Keywords:** social networking, adolescents, communication, motives of use, social desirability, gender differences, age differences

## Abstract

The evolution of digital media has changed the patterns and motives for its use among adolescents and has impacted their communication choices within their family and social networks. The objectives of this study are to understand whether peers communicate through a social network (SN) or by voice and their view of the relative social desirability of these alternatives. After the informant’s consent signature, adolescents completed a series of self-report questionnaires on the use of SN, communication preferences, and social desirability online. Most of the adolescents belonged to the 17–19 age group (83.6%) and were female (68.9%). Adolescents spent more than 3 h/day on Whatsapp and more than 2 h/day on Instagram, while the use of Facebook was on average only 35 min/day. Females used digital media longer than males. Adolescents aged 17–19 years choose more Facebook and voice modes compared to adolescents aged 14 and 16 years. Alternative modes of Whatsapp and voice were chosen more than social networks in their communication strategies, especially for negative topics. Motives for use were, in addition to boredom, related to maintaining one’s social sphere with peers. Some educative considerations were made based on these results.

## 1. Introduction

During the past decade, the use of interactive digital and social networks has grown significantly, and current research studies have evidenced both benefits and risks to health in teenagers. Health benefits may include enhancing access to valuable support networks and promoting health behaviors and social inclusion [1,2]. Negative effects could be increasing dependence behaviors, including substance abuse, sexting, or Internet-addiction risks [3,4].

The evolution of digital media has also changed the patterns of use, the motives of use, and the impact on their communication choices in their social and family networks.

But how are digital media used among teens today, and which modes of use are the most popular?

According to an annual report on teenagers belonging to two age groups, 15–17 and 18–19 years, young people who use the Internet and who, above all, use it every day are constantly increasing year after year.

Internet use increases with increasing age, and it is estimated that the age range engaging in maximum use is between 15 and 17 years, with 93.5% and 94.2%, respectively, of connected females and males [5]. However, the percentages are increasing.

There is an increase in the percentage of people who use the Internet daily (15–17 years 83.7% vs. 86.8%; 18–19 years 87% vs. 88.2%; 20–24 years 86% vs. 87.2%) [6].

The most recent data provided by the July 2021 report from We Are Social and Hootsuite show that there are 4.48 billion users of social networks active in all the world, or about 57% of the world population, with an increase of 13% compared to the previous year. Data from 18 July 2021 show how Facebook is the platform with the highest number of users, followed by YouTube, WhatsApp, and Instagram. TikTok is the seventh. Today, there is also a growth in the use of HVSM (Instagram) compared to Facebook, without gender differences [7].

Given that there seems to be a normative difference in the expression of positive and negative emotions through new digital media [8], we wondered which were the preferred communication modalities of adolescents in different situations, if these trends changed in the case where one discussed positive or negative questions, and if gender and age somehow influence such communication choices. In this regard, a general preference of social networks is envisaged for positive topics, in particular Instagram [8], and show that gender differences in the use of social media are decreasing [7]. 

Another study that focused on motivations for the use of social networks (SNS) identified group belonging, collective self-esteem, and gender effects as the main reasons among older adolescents. Females were more likely to report high collective positive self-esteem, greater overall use, and SNS use to communicate with peers. Females also posted higher means for grouping, passing time, and entertainment. Males were more likely than females to report negative collective self-esteem and the use of SNS for social compensation and social identity gratifications [9].

The continuous advancement of digitalization exposes the social identity of young people to an ever-increasing digital mediation. Studies on body image and internalization processes [10] indicate a greater social comparison mediated by the new means of communication, as they allow us to follow idealized models from all over the world. In this sense, adolescents could show high levels of social desirability due to the great connection they have with the world through continuous social comparisons mediated by social networks and other digital media. They find themselves more than ever in a social environment that exposes them to judgment and comparison to others [11].

With the development of different social media sources, interpersonal communication is really changing, and new communication skills are involved [12]. Mobile phones and instant messengers, such as Whatsapp, together with social network use (i.e., Facebook and Instagram) allow to communicate with distant people, to give a built self-image coherent or not coherent with the true self, and use pictures and photos to narrate their everyday life. People interact by mixing these two different communication types in everyday life: “oldest” face-to-face (FtF) communication and “newest” thorough interpersonal media that have expanded the territories of mediated interactions. 

Social interactions include both direct and mediated communication. However, each type of communication has different characteristics. Mediated communication is maintained using the functions of interpersonal media mode, while FtF communication is performed in a natural setting without any technological support. The results of a study proposed a model [12] on the key role of mediated interpersonal communication in the development of communication competence in relationships. This study revealed that communication competence was positively associated with mediated interpersonal communication competence. The indirect effects of communication competence through media efficacy and social presence were empirically supported. Maintenance of relationships was found to mediate the effect of communication competence on mediated interpersonal communication competence. 

### Research Questions

The main objective of this study is to understand whether everyday life is somewhat maintained in its old communication rules and settings when adolescents want to communicate their daily routines to others. 

1. The first objective is to understand how peers communicate with each other today. Specifically, we aimed to know:

1a. How much time did they spend in SNS activities? Were there gender or age differences in SNS activities?

1b. Which means of communication are preferred by today’s teenagers to talk about certain topics?

1c. Is there a significant difference in frequency of use when comparing social or nonsocial communication choices? Is the choice to use social networks (Facebook and Instagram) or to use other direct sources (Whatsapp and voice conversations) mediated by some sociodemographic variables, such as gender or age?

1d. What about the motives of the use of every communication device?

2. The second objective is to glean information on how much adolescents try to be socially desirable and what consequences this construct could have on their use of SNS. Specifically, our objective was to answer these questions:

2a. Are adolescents screened by a level of social desirability (low, medium, high)?

2b. Is social desirability influenced by the age or sex of adolescents?

## 2. Materials and Methods

### 2.1. Procedure

After completing the digital version of the questionnaire, built with the use of GoogleForms, it was passed to the recruitment phase of the participants, i.e., adolescents between the ages of 14 and 19. The procedure used for the recruitment of the research participants was that of a snowball. This method makes it possible to use digital means and word-to-word communication between people and reach as many contacts as possible.

For each communication and message, after making sure that the teens contacted were interested in the research, the procedure consisted of explaining the terms and purposes of the research as the administration modalities. For teens under 18 years of age, we obtained the signed parental consent form, while for teens over 18 years of age, they directly signed the online consent form. The next step was to contact the various associations and local authorities that, by virtue of their activities, could facilitate sample research: schools, association groups, and peer networks in Emilia Romagna region, Italy.

The search for the sample and the administration lasted a total of two and a half months, in which the final number was reached (*n* = 347). Of the 400 total children contacted, 347 completed the questionnaire and participated in the research. The response frequency was therefore 86.75%.

### 2.2. Participants

Table 1 shows the characteristics of the participants. Most of the adolescents belong to the age group 17–19 years old.

The main sociodemographic data collected on the characteristics of the participants were: sex, age, number of siblings, marital status of the parents, and the school year attended (see Table 1). 

Age was divided into two age groups, 14–16 years (attending the first three years of high school) and 17–19 years (attending the other last two years of high school). The number of siblings was categorized into none and more than 2. The marital status of the parents was divided into married, cohabiting, separated/divorced, and one parent. Since the sample consisted of only two middle school boys, it was decided to exclude this category from the sample and report only the participants attending high school.

Most of the sample comes from two high schools in the province of Modena, Emilia Romagna region. Furthermore, the sample has a higher concentration of female gender than male gender and a higher frequency of adolescents in the age group 17–19 years old.

### 2.3. Instruments

#### 2.3.1. SNS Time Using

To understand how long teens thought about using the social network and the proposed instant messaging system, we were inspired by the work of Pempek and McDaniel (2016). We then calculated the frequency of use with a 9-point scale proposed for the estimate of frequency of use for Instagram, Facebook, and Whatsapp; that is, how much time do you spend on Instagram?

(1) Never
(2) 10–30 min a day
(3) 31 min–1 h a day
(4) From 1 h and a half to 2 h a day
(5) From 2 h to 3 h a day
(6) From 3 h and a half to 4 h a day
(7) From 4 to 6 h a day
(8) More than 7 h a day

With this questionnaire, we can obtain information on the frequency of use of different social networks or instant messaging. These time bands will then also be reduced to average quantities in minutes of use to make it a point variable of an independent type with the other variables of interest in the study.

#### 2.3.2. Motivations to Use Facebook

To get an idea of the reasons why teens use Facebook and other social networks, a simplified and adapted version of the Motives to use Facebook [13,14] was used. The original scale was made up of 20 items, each representing a possible motivation for the use of Facebook, evaluated with a 5-point Likert scale.

In this study, it was decided to adapt the questionnaire to a more generic study context and not just to Facebook and to resize it. The final product was a 20-element checklist. To not weigh down the commitment burden of adolescents with too many answers, it was decided to set the number of answers to the three main reasons they used social networks, without information on the frequency of choice of the reason of use. However, teens were left with the freedom to indicate more than 3 answers, so the selection was anchored in the 3-element checklist, and the too-selectable answers were limited.

It was also preferred to add another item that allowed the sample to personally write a reason to use social networks if it was not included in the list provided by the questionnaire.

#### 2.3.3. The Marlowe—Crowne Social Desirability Scale (MCDS)

The MC-SDS is a questionnaire composed of 33 items with a dichotomous answer (true or false), concerning behaviors that adolescents could implement in everyday life [15]. Eighteen of these items refer to socially accepted but unlikely behaviors that indicate a compiler’s need to give a positive self-image and were counted with 1 point for each item. The remaining 15 instead focus on more common but not shared social behaviors. Some examples of these items are as follows: *“There have been times when I felt like rebelling against people in authority even though I knew they were right,” or “It’s sometimes hard for me to carry on with my work if I’m not encouraged,”or again “No matter who I’m talking to, I’m always a good listener.”*

The disingenuous choice among these items indicates a propensity of the compiler to want to hide normal behaviors in order not to appear as socially undesirable, obtaining 1 point for each answer. In this way, higher scores will correspond to a greater need to hide their socially unwelcome behavior and the desire to show socially accepted behaviors instead to appear more desirable. The items must then be divided according to where the user should answer TRUE or where the user should instead reply FALSE. The total score is found from the sum of the true statements and could indicate low (0–8), average (9–19), or high scorers (20–33). For Cronbach’s alpha, answers for TRUE were (α = 0.57) and for FALSE responses were (α = 0.63).

A high number of socially desirable responses might indicate that the respondent is generally concerned with social approval and conforming to societal conventions, while a low score might indicate that the respondent is less concerned with such things and is more willing to answer survey questions truthfully and represent themselves accurately. 

However, Crowne noted that the motive to answer in socially desirable ways is more than a simple need for approval; it also entails a repressive defense against a vulnerable self-esteem.

#### 2.3.4. “Tell Me How You Communicate and I Will Tell You Who You Are”, List of Communication Preferences 

This tool was created ad hoc to understand the communicative preferences of children in various fields and contexts proposed [14]. The very short questionnaire aims to evaluate the methods preferred by young people to communicate with others on certain topics.

Each question (item) corresponded to a sphere of interest (i.e., family, friends, etc.), positive or negative, and a checklist of answers that included 4 different modes of communication and an additional answer in which the participant could write other possibilities. 

The items included in each of these selected spheres of interest are the following:

(1) Family: 3 items, including 2 negative and one positive.
(2) Friends: 2 items, namely one positive and one negative.
(3) School: 2 items, including one positive and one negative.
(4) Special person: 2 items, including one positive and one negative.
(5) Other: Three negative items, namely one dedicated to teasing, one concerning loneliness, and the last concerning mood or feeling down.

For each proposed situation, the sample was asked to select which modes of communication they preferred and then which ones they considered most appropriate between:

(A) Instagram: Representative of the social-network category of HVSM (α = 0.75).
(B) Facebook: Representative of the classic social network category (α = 0.68).
(C) Whatsapp: Representative of the verbal instant messaging category (α = 0.87).
(D) A voice: Generic representative of classical oral speech (α = 0.85).

Furthermore, an option was given that allowed children to write or directly integrate solutions that they preferred but that were not present in previous answers.

With these questions, we wanted to know between the different areas (1, 2, 3, 4, 5) and divided by type of content (positive or negative), which communication channels were preferred by adolescents (A, B, C, D, E).

### 2.4. Statistical Analysis Plan

The plan of statistical analyses carried out will now be shown, point by point.

(A) Statistical analysis on the daily time spent on Facebook, Instagram, and Whatsapp: To facilitate the analysis, it was decided to transform the measurements of time spent on digital media (see frequency of use, previous page) into a specific variable, transforming each band into its equivalent in minutes, as follows:

A1—Descriptive statistics of time in bands and its translation in minutes, with average values and standard deviations. The results of the *t*-tests are shown using the variable of time spent on different digital media, with time in minutes and with gender and age (14–16 years, 17–19 years) as independent variables.

A2—Descriptive statistics regarding communication preferences. The results obtained are indicated by topic (family, friends, school, special person, themselves) and by response (Facebook, Instagram, Whatsapp, by voice, other). The results of five different tests are reported. The first test compares the answers grouped by type of answer for each topic (e.g., the average number of Instagram respondents in the family topic) with the two different age groups examined. The second *t*-test considers the communicative channel grouped by positive arguments (e.g., those who have chosen Instagram for positive topics) and those who have selected the same type of communication choice but in negative situations (e.g., average number of Instagram selections for negative topics). The third *t-*test sees the dependent variable as the average number of total responses assigned to Instagram and as a gender-dependent variable. The fourth *t-*test investigates the same average of responses for Instagram but using the age groups as a dependent variable. The fifth *t-*test compares the average responses obtained from the grouping according to the selected and positive/negative communicative modality (e.g., the average response for Instagram in positive subjects) and age groups (independent variable).

A3—By grouping the social communication preferences (Facebook and Instagram choices) and nonsocial (voice and Whatsapp) choices, a series of correlations with Pearson coefficient was made.

A4—Descriptive statistics for the checklist on the reasons for using social networks are indicated. 

(B) Analysis of social desirability. 

B1—The results obtained from the social desirability test were also grouped into 3 bands, namely low, middle, and high, to carry out further correlations and analyses.

B2—The results of two *t-*tests are presented with social desirability as a dependent variable. In the first test, we present the results of the gender and in the second those of the age groups.

## 3. Results

### Statistical Analysis of the Daily Time Spent on Facebook, Instagram, and Whatsapp (A)

A1—Social time use and differences between age and sex

The time measurement was initially performed based on the time bands defined by the questionnaire and then transformed the results obtained into daily minutes, a type of punctual variable considered more useful in subsequent measurements (Table 2).

An independent sample *t*-test was performed by entering as a dependent variable the time spent on Whatsapp, Instagram, and Facebook in minutes and with gender as independent. The difference between the averages considering gender was revealed as significant for both the time of use of Whatsapp and that of Instagram, while for Facebook, the result was not significant (Table 3 and Table 4).

To understand any differences between the time spent on digital media according to the age of the adolescent, a further *t*-test was carried out between the time measured in minutes along the two age groups investigated (14–16 and 17–19). The results reported a significant difference between the averages only in terms of time spent on Facebook (*t* (342) = −3.8; *p* = 0.0001), with boys in the 17–19 age group using this platform more (Mean = 47.76; SD = 76.85) than those in the 14–16 age group. (Mean = 19.42; SD = 54.01).

A2—Communication preferences: descriptive statistics, possible differences along age and gender, and prevalence of a social or a nonsocial communication style.

The results of the communication preferences questionnaire are reported for each topic, and the answer is shown in Table 5. For reference, each item is listed with the corresponding code used during the analyses:

- SC1 = school positive, “When I get a good grade at school, I feel like talking about it”
- SC2 = family negative, “When my family makes me angry…”
- SC3 = positive for friends, “When my friends make me have fun…”
- SC4 = school negative, “When I get a bad grade at school…”
- SC5 = negative for the special person, “when the person I like makes me sad…”
- SC6 = family positive, “When my family makes me happy…”
- SC7 = negative self, “When some people make fun of me…”
- SC8 = positive for special people, “when the person I like makes me happy…”
- SC9 = family negative, “When I argue or argue with my parents…”
- SC10 = friends-negative, “When my friends make me angry…”
- SC11 = negative self, “When I feel alone…”
- SC12 = negative self, “When I feel down…”

The results obtained were then grouped by topic (family, friends, etc.) and by communication choice. In this way, there was a frequency of how many had selected each communicative choice for each topic, and the age band was inserted as an independent variable to understand if it had any kind of effect (Table 6). 

A paired-sample *t*-test was performed in which the communication methods divided among themselves for positive or negative arguments were compared. In this way, it was possible to understand whether each communicative choice was selected on average more for positive or negative arguments. The results are shown in Table 7.

We then wanted to investigate whether the age range of adolescents (14–16, 17–19) could influence the choice of communication for both positive and negative arguments. From the results of the *t*-test for independent samples, only the use of the voice mode for negative arguments was significant (*t* (342) = −3.07, *p* < 0.05). From the compared averages, it was understood that adolescents aged 17–19 years more significantly chose the by-voice communication modality (M = 5.06, SD = 2.03) compared to those aged 14–16 years (M = 4.34, DS = 2.3).

Then, it was investigated whether the choice of Instagram was related to gender. A *t*-test was then performed using gender as an independent variable and Instagram’s average of choices as a dependent variable, without differentiation by topic. The *t*-test in the means reported a significant difference (*t* (342) = −3.85, *p* < 0.05). From the averages, it was found that female participants (M = 1.87, DS = 2.1) choose Instagram more times than male participants (M = 0.99, DS = 1.58).

A3—Analysis on the choice to communicate on various topics using a social network (Facebook or Instagram) or other nonsocial methods (Whatsapp and by voice).

A series of *t*-tests for the paired samples was performed to verify the significance between the means of the pairs examined. Pairs 1, 2, and 3 resulted as significant. Table 8 and Table 9 show the results.

A4—Reasons why adolescents use social networks

The descriptive statistics carried out on the checklist regarding the reasons why social networks are used showed the following main ones in order of frequency: to spend time when I am bored (50.10%); to communicate with my friends (48.10%); to send a message to one of my friends (41.50%); to maintain contacts with my friends (39.50%); to look at the photos/stories of others (36.90%); and to be able to communicate with people who are difficult to reach (29.7%).

B1—Frequencies on social desirability

The adolescents obtained social desirability scores that were grouped into three bands: low (6.9%), middle (74.6%), and high (17.6%).

B2—Gender and age differences in social desirability scores

To understand whether age or gender were related to these results, two *t*-tests were performed that scored desirability as a dependent variable and gender and age groups as independent variables. Sex was not significant (*t* (342) = 0.1 and *p* > 0.05). On the other hand, the age was significant (*t* (342) = 1.97, *p* = 0.49). Specifically, adolescents aged 14–16 years (M = 15.96) had a significantly higher average social desirability score than those aged 17–19 years (M = 14.62).

B3—Relationships between the level of social desirability and SNS use

Pearson’s correlations were run to identify possible significant associations between social desirability and the time/type of use of the various social platforms. Table 10 shows only the significant correlations.

## 4. Discussion

The first objective that motivated this study was to know and understand more, year after year, about the generations of adolescents who follow social networks in Italian schools. Following an educational point of view, it is necessary to understand the needs and preferences of social interactions on the Web and implement any psychoeducational interventions. However, an extremely current problem is the intrinsic mutability of today’s adolescents, particularly in the communicative and social field. The new available technologies are changing the world as we know it [16] in all aspects. Change and globalization are reaching everyone and, similarly to the technology they bring, are without time or boundaries. 

While today’s adults can watch and observe changes by paying attention to how things were before they happened, today’s teenagers find themselves growing and absorbing the laws of a world that makes immediate technological-communicative evolution a founding characteristic in a context of continuous change.

The first research question of this work moves precisely in this sense, asking how to communicate today’s adolescents, with what tools, and for how long. The data obtained will serve to build a snapshot of a generation in constant change, and it is necessary to understand and study from moment to moment.

In this sense, data lead us to a continuous growth, both in the amount of time used day after day on the Internet [6] and in the use of social networks and digital media, especially by those who access from mobile devices [7]. Teens in our study seem to partially confirm this trend, especially by differentiating the type of digital medium. In this sense, they claim to spend more than 3 h on average per day on Whatsapp and more than 2 h a day on Instagram, while the use of Facebook stands at an average of only 35 min per day. Data related to the time spent daily on Whatsapp and Instagram of children participating in our study tended to be higher than the national average of Italy, which is around 2 h per day using social networks as a whole [7].

From the results obtained, it can also be stated that the women in the sample use all three digital media for a longer time each day than the men.

Instagram and Whatsapp are used nearly two hours a day more by women than by men. However, age does not seem to affect Instagram and Whatsapp, as the only difference is found for Facebook, which is used more with increasing age. This trend is confirmed by some studies [7,10] where Whatsapp is the first medium used, Instagram follows and stands as a rapidly growing social network [7], while Facebook seems not to be used much until late adolescence.

The present study aims to understand in which circumstances or situations social networks and instant messaging are used and chosen as relevant media. Regarding age, adolescents aged 17–19 years seem to choose and to use Facebook and voice modes more on average compared to adolescents aged 14 and 16 years. The voice mode, in particular, is also significant for talking about yourself and a special person with increasing age. All this can be circumscribed within the normal evolutionary framework of the adolescent, who, growing up and approaching adulthood, will have a greater share of opportunities to talk and discuss private topics, such as those regarding family, loved ones, and problems related to themselves. On the other hand, they will be able to make better use of their cognitive abilities to speak verbally about topics that, as they grow up, they will tackle more confidently.

While Instagram and voice mode are preferably chosen for positive arguments, Whatsapp seems to be preferred more than anything else for negative arguments. Therefore, the trend indicated by Waterloo et al. [8] is therefore maintained, concerning the expression of emotions on various social networks, where Instagram is confirmed as more suitable for positive emotions and WhatsApp also for the expression of negative emotions. This also confirms the inherent difference between these two means of communication since Instagram, known as HVSM [10], becomes a social laboratory in which to show oneself and show what one prefers. Whatsapp, being instant messaging, instead guarantees greater confidentiality and anonymity to conversation, which will probably also concern negative things that maybe one would not want to discuss publicly.

The oral communication method was also confirmed once again, on average, to be used more, this time for negative arguments, in the 17–19 age sample than in younger teenagers. This result aligns with what has been said about the importance of age with respect to voice communication since by increasing age, one is probably more able or confident to speak out about negative arguments than when one are younger.

Then, it was assumed, based on European data [7], that there are no significant gender differences in the use of Instagram. However, the hypothesis was disconfirmed: females seem to choose Instagram more as a communicative mode than males. This means that the females of the sample use Instagram more often a day, but they also choose it significantly more times as a way of communicating than do the males. In general, the time spent on Instagram was also positively associated with the choice of the same as a communication mode in all participating adolescents.

Other information needed to be understood and studied on today’s teenagers deals with proper use of social networks as a whole as a means of communication compared to the different other modes, e.g., analog as well as digital. This is to understand the proportion of the phenomenon in the adolescents participating in the study and then to deepen the further analysis and to see if these choices affect growth. In general, the choice of social networks (Instagram and Facebook) was on average lower than the choice of the other modes (Whatsapp and by voice). Such a preference for communication methods alternatives to social networks is also reflected in the averages of the individual topics: for any topic investigated in the study (e.g., family, friends, oneself, special person, and school), Whatsapp and voice alternative modes were chosen more than social media network.

For all the topics covered, we wanted to investigate if there was a situation in which Instagram was chosen over Whatsapp and by-voice methods. The results reported averages statistically superior to Whatsapp and verbal methods for Instagram for each proposed topic.

For all arguments, in fact, women are the ones who use on average both Instagram and Whatsapp more than men, further confirming the afore-mentioned discussion. Additionally, regarding the use of Instagram for positive or negative issues and Whatsapp for negative issues, women maintain a higher average use than their male counterparts. 

Such a result leads us to ask ourselves why this happens, and more generally, what are the reasons that guide or orient the use of social networks? In this regard, 50% of the sample reported using social networks to pass time when bored, 48.1% to communicate with their friends, and 41.5% to send a message to a friend. Furthermore, 39.5% said they use social media to stay in touch with their friends and 36.9% to look at the photos/stories of others. Aside from boredom, three of the most chosen reasons relate to maintaining one’s social sphere with peers. For teenagers, this feature, accompanied by an investment of energy and interest in the peer group, is fundamental for growth and evolution towards adulthood [5,17,18]. However, the comparison with peers is now also taking place through the use of digital media but often to maintain and to rework existing relationships even in real life.

The group of study participants fell for the most part (74.6%) in the middle range of social desirability, while 6.9% were in the low range and 17.6% in the high range. It therefore appears that, excluding those who are positioned in the middle range, the adolescents of the sample present more levels of social desirability than low; in a certain sense, they want to be more deemed desirable. These data confirm what has been hypothesized for two main reasons:

First, social desirability is a construct that lends itself particularly well to adolescent age development. In fact, adolescents want to be appreciated and recognized by peers in order to acquire the necessary self-confidence that will facilitate the resolution of development tasks. However, what is worrying is that this normal need is excessively accentuated, giving the adolescent a disproportionate need to appear desirable, especially in the context of a social showcase, such as social networks [11].

Such exposure and need for desirability could also be related to body-image management issues as well as internalization processes [19] or dependence on the Internet and social networks [2]. From the sample examined in the present study, there appear to be no specific differences in social desirability related to the sex or marital status of the parents but related to age with an average significantly higher social desirability for younger adolescents aged 14–16 compared to older ones aged 17–19. This can therefore be interpreted as a confirmation of the fact that social desirability is a phenomenon that particularly affects adolescents at the beginning of adolescence but that with growth, these values normalize as one approaches complete maturity.

Social networks can perhaps be understood as a virtual laboratory of one’s social identity, particularly used by those who feel more than others the need to appear desirable. Despite this result, social desirability was negatively associated with the time spent on Instagram. Likewise, the social desirability score was negatively associated with the choice of using social media generically and specifically with topics relating to school and friends. According to the results obtained, there is a countertrend that darkens or numbs the role of social desirability in mediating the relationship with social networks. One can perhaps think that the increased need to appear desirable not only leads to changes in the quantitative use of social networks but also modifies this phenomenon qualitatively, perhaps leading to different uses not strictly related to the time spent there or the choice to use them as means of communication. Further studies in this regard may probably clarify this phenomenon by using other measurements as well.

### Strengths and Limits

Undoubtedly, the strength and weakness of this work is the research sample. Although the overall participants are a notable number, the sample is not very balanced, with a majority in the older age group (17–19) compared to those who placed in the youngest group (14–16). In addition, women were more numerous than men. Another limit of this work concerns the values of social desirability. As all the questionnaires proposed were of the self-report type, social desirability will influence the results, as we have seen especially in general models. As stated several times during the discussion, social desirability is, however, a necessary and important measurement, especially as regards adolescence; this makes it a construct that can disturb the results as much as enrich them. We must note also that the scales of the social desirability scores obtained a moderate level of reliability, and the results related to this construct should be taken with caution.

A further limitation is the lack of balance between the items of the communication choices; in this sense, it would be desirable to increase the total number of items and match arguments that possess only negative items and lack positive items. 

Despite these limitations, the work boasts a multidimensional approach, which made it possible to enrich information concerning, on the one hand, the state of adolescent well-being and self-efficacy and, on the other, to obtain important information regarding their communication preferences, the reasons behind them leading to the use of social networks, and the different norms and influences these constructs have measured. 

A desirable future prospect is to expand this type of research and focus more on the opportunities that teens can and should take advantage of the world around them. The ambiguity of the results in the field of the use of social networks shows one direction of investigation in which it is necessary to provide further clarity and, above all, to continuously update the results in line with the sudden socio-technological changes of the current modern period. Therefore, it is good to deepen knowledge both of context and of practices and norms that are established when part of the social sphere of an adolescent involves digitizing and grasping critical issues, developments, and opportunities. Another study could be concerned with the role of parents and friends in the use of social networks since studies on the subject [20] have underlined its importance.

## 5. Conclusions

Today’s teenagers, while spending many hours a day on social media, can choose what topic to communicate through them and what to express face to face. There are some main conclusions that we can draw from this work.

First, it must be accepted that social networks, and the digital in general, are the present and the future of the inhabitants of this planet in the field of communication, and not only is it not good practice to demonize them, looking only at their defects and risk factors without considering their positive properties and the evolution they represent in mass communication, but it is also not useful to demonize the use that new generations make for them or the amount of time spent on them. It is much more productive to try to understand how social networks work and how they are used by pre-adolescents and adolescents who will soon be adults, to sensitize them to the responsible use of them, and to recognize their dangers but also to be able to learn ourselves from those who have almost grown up with this technology. Social networks are not harmful, but their use can be, and for this reason, they must be viewed without fear and suspicion but with curiosity, understanding, and a healthy critical spirit.

The purpose of this work was to take stock of the situation and propose a positive vision of adolescents of this generation, who have not stopped feeling emotions to submit to the control of digital platforms but have learned to transpose their feelings within them. They consider social networks as means of communication, without forgetting the real world and its role within it. The hope is that we have managed to communicate this message with the hope that it will be the basis for a better understanding of the role that social networks play in all of our lives.

## Figures and Tables

**Table 1:** Sociodemographic characteristics of the participants. The numbers shown in parentheses indicate the ordinal level of variables.

		Frequency	Percentage (%)	Mean	SD
Gender	Male (1)	105	30.3	1.69	0.46
	Female (2)	239	68.9		
Age groups	14–16 years old (1)	54	15.6	1.84	0.36
	17–19 years old (2)	290	83.6		
Number of siblings	0 (0)	56	16.1	1.28	0.89
	1 (1)	180	51.9		
	2 (2)	63	18.2		
	>2 (3)	45	13.0		
Parental Relationship status	Married (1)	247	71.2	1.57	0.95
	Cohabitants (2)	11	3.2		
	Separated/divorced (3)	73	21.0		
	Only one parent (4)	13	3.7		
Level of education in secondary school	1° (1)	32	9.2	3.54	1.25
	2° (2)	39	11.2		
	3° (3)	76	21.9		
	4° (4)	104	30.0		
	5° (5)	91	26.2		

**Table 2:** Mean and standard deviation of several social networks time use by time bands or overall minutes (min).

		Mean	SD
Time bands	Facebook	1.13	1.46
	Instagram	3.48	2.10
	Whatsapp	4.0	1.94
Time in minutes	Whatsapp (min)	181.17	131.58
	Facebook (min)	35.49	69.2
	Instagram (min)	153.18	126.23

Legend: (1) Never; (2) 10–30 min a day; (3) 31 min–1 h a day; (4) From 1 h and a half to 2 h a day; (5) From 2 h to 3 h a day; (6) From 3 h and a half to 4 h a day; (7) From 4 to 6 h a day; (8) More than 7 h a day.

**Table 3:** Independent samples *t*-test: gender and time spent on each social network.

	*t*	df	*p*
Time in minutes on Whatsapp	−7.6	342	0.0001
Time in minutes on Instagram	−6.86	342	0.0001

**Table 4:** Group descriptive statistics.

	Gender	*n*	Mean	SD
Time in minutes, Whatsapp	male	105	105.71	85.59
	female	239	214.33	134.66
Time in minutes, Facebook	male	105	32.28	62.87
	female	239	36.9	71.97
Time in minutes, Instagram	male	105	87.00	86.82
	female	239	182.25	129.95

**Table 5:** Frequencies of communication choices.

		Frequencies	Percentage (%)
SC1	Instagram	78	22.5
	Facebook	0	0
	Whatsapp	141	40.6
	Voice	252	72.6
SC2	Instagram	6	1.7
	Facebook	2	0.6
	Whatsapp	146	42.1
	Voice	214	61.7
SC3	Instagram	142	40.9
	Facebook	10	2.9
	Whatsapp	118	34
	Voice	206	59.4
SC4	Instagram	21	6.1
	Facebook	0	0
	Whatsapp	116	33.4
	Voice	236	68
SC5	Instagram	39	11.2
	Facebook	5	1.4
	Whatsapp	156	45
	Voice	229	66
SC6	Instagram	56	16.1
	Facebook	4	1.2
	Whatsapp	115	33.1
	Voice	254	73.2
SC7	Instagram	17	4.9
	Facebook	3	0.9
	Whatsapp	115	33.1
	Voice	226	65.1
SC8	Instagram	101	29.1
	Facebook	7	2
	Whatsapp	174	50.1
	Voice	251	72.3
SC9	Instagram	1	0.3
	Facebook	2	0.6
	Whatsapp	125	36
	Voice	256	73.8
SC10	Instagram	18	5.2
	Facebook	2	0.6
	Whatsapp	146	42.1
	Voice	271	78.1
SC11	Instagram	45	13
	Facebook	6	1.7
	Whatsapp	173	49.9
	Voice	174	50.1
SC12	Instagram	28	8.1
	Facebook	4	1.2
	Whatsapp	160	46.1
	Voice	224	64.6

**Table 6:** Communication choice for several topics in the age bands.

	Age Bands	*t*	df	*p*	Mean	SD
Voice Family	14–16 17–19	3.3	342	0.001	1.44 1.73	0.84 0.76
Voice Special person	14–16 17–19	2.37	342	0.02	0.94 1.09	0.6 0.6
Voice Themselves	14–16 17–19	2.23	342	0.02	0.74 0.9	0.67 0.63

Legend: The mean refers to the average number of responses for “voice” in various communicative topics (family, special person, themselves).

**Table 7:** Communicative choice for positive versus negative topics, adopting the paired sample *t*-test.

	Topics	*t*	df	*p*	Mean	SD
Facebook	Positive	0.85	346	0.39	0.04	0.23
	Negative				0.05	0.31
Whatsapp	Positive	16.52	346	0.0001	1.2	1.14
	Negative				2.8	2.41
Voice	Positive	24.9	346	0.0001	2.2	1.1
	Negative				4.7	2.2
Instagram	Positive	14.17	346	0.0001	0.27	0.31
	Negative				0.06	0.13

Legend: Mean referred to the average time that adolescents and young adults declared choosing a specific platform to communicate positive or negative topics.

**Table 8:** *T*-test for paired samples between social and nonsocial means.

		*t*	df	*p*
Couple 1	Social sum utilization—NoSocial sum utilization	39.32	346	0.0001
Couple 2	Social School—NoSocial School	28.27	346	0.0001
Couple 3	Social Family—NoSocial Family	34.84	346	0.0001
Couple 4	Social Friends—NoSocial Friends	25.04	346	0.0001
Couple 5	Social Special Person—NoSocial Special Person	26.76	346	0.0001
Couple 6	Social Themselves—NoSocial Themselves	25.15	346	0.0001

Legend: NoSocial = choices characterized by Whatsapp or voice. Topic = school, family, friends, special person, themselves.

**Table 9:** Descriptive statistics of social and non-social sums.

		Media	*n*	DS
Couple 1	Sum social utilization	1.72 12.9	347 347	2.13 5.3
	NoSocial utilization			
Couple 2	Social school	0.25	347	0.46
	NoSocial school	1.63	347	0.84
Couple 3	Social Family	0.19	347	0.45
	NoSocial Family	0.46	347	1.18
Couple 4	Social Friends	0.51	347	0.6
	NoSocial Friends	2.31	347	1.15
Couple 5	Social Special Person	0.28	347	0.51
	NoSocial Special Person	1.72	347	0.9
Couple 6	Social themselves	0.19	347	0.44
	NoSocial Themselves	1.55	347	0.91

**Table 10:** Pearson’s correlations between social desirability and the use of SNS.

	Instagram Time of Use	Social Sum Choices (Instagram and Facebook)	Use of Social in the Friends Communication Topic	Use of Social in the School Communication Topic
Social desirability	r = −0.26 **	r = −0.14 **	r = −0.19 **	r = −0.14 **
	*p* = 0.0001	*p* = 0.008	*p* = 0.0001	*p* = 0.0001
	*n* = 344	*n* = 344	*n* = 344	*n* = 344

** *p* < 0.01.

## Data Availability

Data could be required to the corresponding author.
